# The role of timing in the development and evolution of the limb

**DOI:** 10.3389/fcell.2023.1135519

**Published:** 2023-05-02

**Authors:** Meng Zhu, Clifford J. Tabin

**Affiliations:** Department of Genetics, Blavatnik Institute, Harvard Medical School, Boston, MA, United States

**Keywords:** limb development, timing and tempo, evolution, developmental biology, morphogenesis and development

## Abstract

The term heterochrony was coined to describe changes in the timing of developmental processes relative to an ancestral state. Limb development is a well-suited system to address the contribution of heterochrony to morphological evolution. We illustrate how timing mechanisms have been used to establish the correct pattern of the limb and provide cases where natural variations in timing have led to changes in limb morphology.

## Introduction

How unique and diverse forms of living creatures evolve is arguably one of the most tantalizing questions in biology. Morphological disparities between the adult forms of related species derive from modifications during embryogenesis. As early as the 19th century, the German biologist Ernest Haeckel postulated two ways by which the modifications could be made in ontogeny to alter the species’ morphology: heterochrony, the displacement of developmental stages in time; and heterotopy, the repositioning of embryonic characters in space (Haeckel, 1875). In an endeavor to couple development and evolution, he proposed the so-called “biogenetic law,” the central argument of which is “ontogeny recapitulates phylogeny,” meaning that the developing embryos of higher animals recapitulate the adult stages of more basally branching animals before developing into their final forms (Haeckel, 1875). Heterochrony in this theory refers to those inconsistencies where the order of developmental events does not obey the sequence of their appearance in evolution. Although the biogenetic law is now discredited, the concept of heterochrony has been preserved in modified form, broadened to include not only alterations in developmental sequence, but also the changes in rate and duration of ontogenetic events (Gould, 1985).

The general concept of heterochrony encompasses a number of discrete mechanisms by which evolutionary change can occur (De Beer, 1951). Today, the most accepted way of breaking down heterochrony posits six distinct mechanisms, placed in two categories, reflecting the polarity of the observed change (Buendia-Monreal and Gillmor, 2018). These include mechanisms that extend development (called peramorphosis) and those that truncate development (called paedomorphosis). The former can be achieved in three ways: processes that follow a normal developmental trajectory but continue for an extended period of time relative to an ancestral organism (hypermorphosis), processes that develop to the same temporal end point, but begin earlier than in the ancestor (pre-displacement), and processes that occur during the same embryonic time period as in the ancestor, but develop at a faster rate (acceleration). Similarly, paedomorphosis can be achieved by three different mechanisms: processes starting at the same time as in an ancestor but developing for a shorter period (progenesis), processes initiating at a later time than in an ancestor but terminating them as normal (pre-displacement), and processes taking place in the same developmental window as in an ancestor but proceeding at a slower rate (neoteny).

These ideas were developed in an attempt to understand morphological evolution prior to modern molecular and genetic insights. Developmental processes, as we understand them now, entail sophisticated interactions between genetic players, themselves expressed at specific times and places, representing a multi-dimensional gene regulatory network. The output of such networks establishes cell behaviors within the embryo, including proliferation, differentiation, migration, and in some cases apoptotosis, which collectively drive morphogenesis. Thus, a molecular understanding of the causality between heterochrony and the evolution of form requires that we comprehend the interplay between genetics and cellular mechanisms in resolving tissue morphogenesis.

Limb development is an excellent system to illustrate the principles by which timing mechanisms are deployed in building tissue and in evolving morphologies. Most vertebrate species have appendages such as fins or limbs. Tetrapod species, the majority of which live on land, have two pairs of limbs, the forelimbs (arms) and hindlimbs (legs). Limb morphologies display remarkable diversity, including differences between the forelimb and hindlimb of a single individual as well as sometimes extreme differences between species. The basic processes required to develop a limb, by contrast, are highly conserved. However, changes in the timing of these fundamental processes have played a major role in the evolution of limbs.

## Limb outgrowth and its timing variations in limb positioning

**FIGURE 1:**
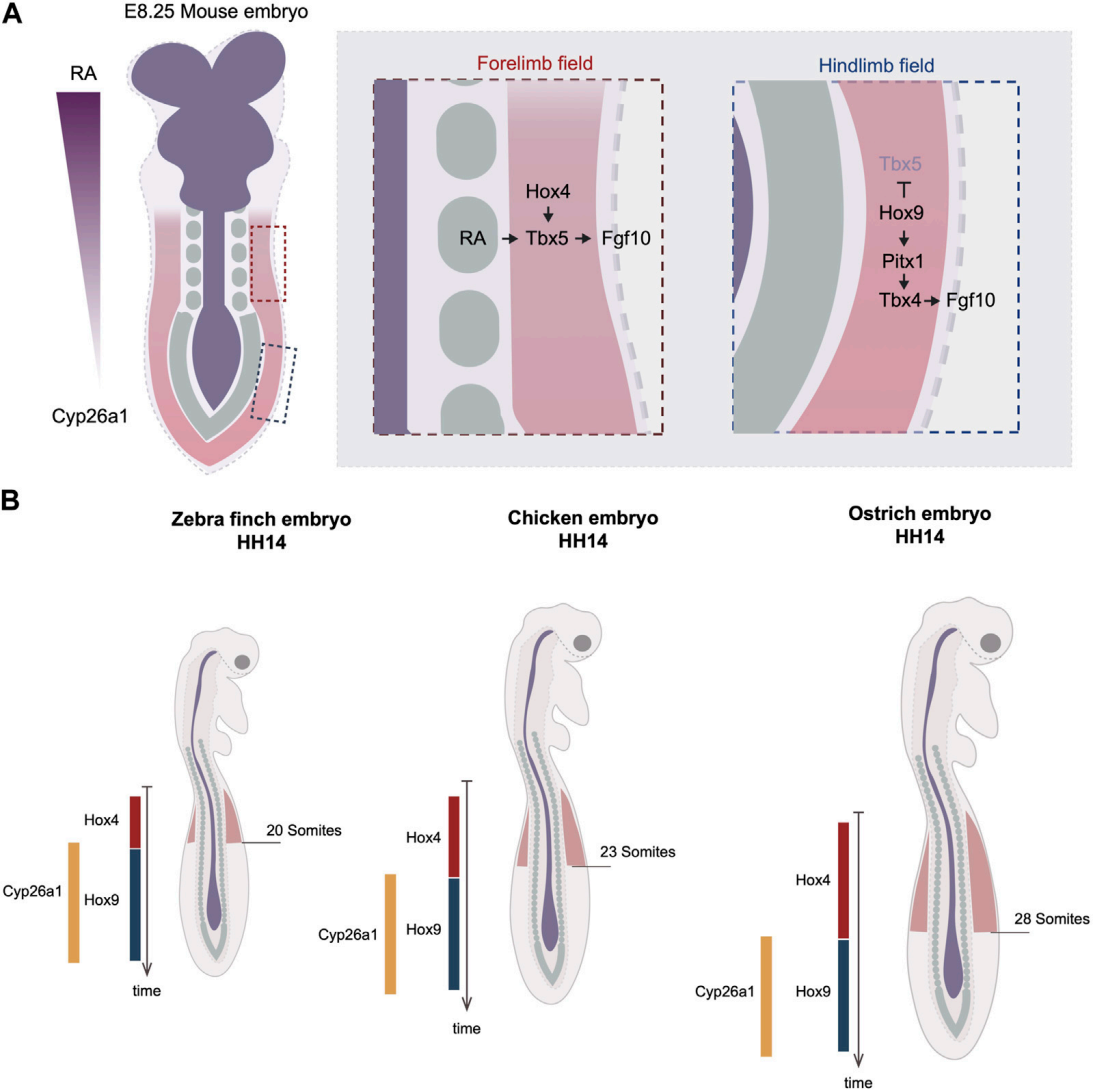
Timing variations in the onset of limb development by the regulation of Hox code. **(A)** Rostral to caudal RA gradient regulates the collinear Hox genes expression at the axial level. In the anterior region, Hox4 specifies the forelimb field by activating the expression of Tbx5. The RA molecules emanating from the trunk can activate Tbx5 in parallel of Hox. In the posterior region, Hox9 antagonizes Tbx5’s expression and induce the expression of Pitx1, which activates Tbx4. Dark blue labels neuroectodermal tissues, green labels somites and red regions level the lateral plate mesoderm. **(B)** The timing of hox genes’ expression varies between three avian species, quail, chicken, and turkey. The delayed termination of Hox4 leads to posterior expansion of forelimb position in turkey. The varied timing of hox genes’ expression is linked to the timing of RA degradation enzyme, Cyp26a1. Red regions indicate forelimb fields.

Development of the limbs begins with the specification of limb fields at the prospective cervical-thoracic and lumbar-sacral vertebral boundaries. Limb-type specific T-box transcription factors, Tbx5 and Tbx4, are expressed in these locations and specify the forelimb and hindlimb fields respectively (Logan et al., 1998; Rodriguez-Esteban et al., 1999; Takeuchi et al., 1999; Naiche and Papaioannou, 2003; Rallis et al., 2003; Duboc et al., 2021) (Figure 1A). These Tbx genes activate an FGF paracrine signaling ligand in the mesoderm, Fgf10. Fgf10 serves both as a morphogenetic cue and as a cytokine; on the one hand it triggers the loss of the epithelial architecture of the LPM, prompting the transition to a mesenchymal state (Gros and Tabin, 2014), and on the other hand it enhances cell proliferation (Sekine et al., 1999). While Fgf10 transcription is restricted to the LPM, secreted FGF10 protein diffuses to the flanking ectodermal tissue, where it activates expression of another FGF ligand, Fgf8 (Ohuchi et al., 1999). The Fgf8-expressing ectodermal region is shaped as a crescent ridge located at the most distal point of the limb bud, named Apical Ectodermal Ridge (AER). Fgf8 travels backward from the AER to the LPM, where it retro-activates Fgf10, forming an Fgf10-Fgf8 positive-feedback loop that effectively sustains limb outgrowth (Xu et al., 1998) (Figure 1A). This gene regulatory module for limb outgrowth is highly conserved in amniote species. However, the timing of the onset of this module is subject to variation, which creates alterations in limb positioning.

### Hox timing and limb positioning

The limb position is often referenced to somite numbers along the rostral to caudal axis of the trunk. The positions of the forelimb and hindlimb vary among different species. For example, the chicken forelimb forms adjacent to somite levels 15–20 and the hindlimb to somite levels 26–32, whereas in the mouse, the forelimb forms adjacent to somite 8–10 and hindlimb adjacent to somite 24–26 (Burke et al., 1995). As somitogenesis occurs parallel to the differentiation of lateral plate mesoderm, the limb position is a result of the relative rates of somitogenesis and limb field specification. During gastrulation, the homeobox (Hox) clusters are expressed in a colinear fashion along the anterior-posterior axis. The Hox genes are composed of four paralogous clusters (HoxA to HoxD). The temporal activation of Hox genes follows the same order as their 3’ to 5’ arrangement in the genome, a phenomenon known as “temporal collinearity,” which, as the Hox expressing region extends posteriorly, generates the sequential array of spatially colinear Hox domains positioned from rostral to caudal of the trunk (Deschamps and Duboule, 2017) (Figure 1A). The expression of these Hox genes in the lateral plate is responsible for establishing the location of the limb fields. The position of the Tbx5-expressing forelimb field overlaps with the expression domains of anterior Hox paralogues 4 and 5, whereas the Tbx4-expressing hindlimb field overlaps with the expression domains of Hox paralogues 8 and 9 (Burke et al., 1995; Minguillon et al., 2012). Moreover, Hoxb4 overexpression is sufficient to induce ectopic Tbx5, whereas the mis-expression of Hoxc9 in the forelimb field can induce ectopic Pitx1, an upstream activator of Tbx4 (Minguillon et al., 2012; Moreau et al., 2019a). Conversely, overexpression of the dominant-negative form of Hoxb4 causes downregulation of Tbx5, whereas the overexpression of the dominant-negative of Hoxb9 leads to the expansion of the forelimb domain (Nishimoto et al., 2014; Moreau et al., 2019a). There is evidence that Tbx5 is, in fact, a direct target of Hox activity. A small genomic region that is localized upstream of Tbx5 intron 2 has been revealed to be able to drive gene expression in a profile mimicking Tbx5. Within this enhancer many Hox binding sites are found. *In vitro* assays suggest that various Hox factors can physically bind to this small enhancer, including not only the anterior Hox4-5 paralogues but also the posterior Hox9 paralogues (Minguillon et al., 2012; Nishimoto et al., 2014). Although the mutation of all Hox binding sites effectively eliminates the enhancer’s activity, the mutation of Hoxc9-associated sites results in a posterior expansion of the enhancer regime, overlapping with the region of the presumptive hindlimb field (Nishimoto et al., 2014). Thus, these results suggest that the anterior restriction of Tbx5 is a result of not only the activation of Hox4-5 genes, but also the inhibitory restriction of Hox9 genes. The inhibitory regulation by Hox9 is further supported by the finding that the ectopic expression of Hoxc9 in the chicken forelimb field effectively blocks Tbx5 expression (Nishimoto et al., 2014). Together, these results from *in vivo* and *in vitro* assays warrant a model whereby a specific set of anterior and posterior Hox codes specify the position of forelimb and hindlimb fields through the regulation of Tbx genes.

### Species-specific differences in Hox timing regulate limb positioning in avian species

The relationship between temporal and spatial Hox collinearity, discussed above, provides a mechanism by which the location of the limb fields could be shifted by heterochronic changes in the timing of Hox gene expression. Recent studies addressed this idea by comparing Hox expression timing and its correlation with limb positions in a range of avian species. Lineage tracing in chicken embryos suggests that the cell populations within in LPM that give rise to the forelimb, interlimb, and hindlimb are determined during gastrulation (Moreau et al., 2019a). The timing when each of these three populations arises correlates precisely with the timing of expression of the corresponding Hox genes. The forelimb positions in zebra finch, chicken, and ostrich embryos end at the 13th, 15th, and 18th vertebrae, respectively (Figure 1B). Importantly, examining the timing of Hoxb4 expression reveals a delay in its termination (hypermorphosis, in heterochronic terms) in ostrich embryos as compared with chicken, correlating with the posterior shift of the location of the limb field.

How might such changes in timing of Hox gene expression be controlled? Anterior Hox gene expression is controlled by retinoic acid (RA) signaling that emanates from the anterior of the trunk. Although RA is capable of travelling long-distances, the posterior of the trunk expresses an RA degradation enzyme Cyp26a1 that restricts RA activity to the anterior. Importantly, the timing of Cyp26a1 expression correlates with the forelimb position of the three species; it appears earliest in the zebra finch, then in chicken, and latest in ostrich (Moreau et al., 2019a) (Figure 1B). It is therefore likely that the RA levels are maintained for different durations in the three species. This results in differences in the time when Hox4 expression terminates and Hox9 expression commences, and hence in distinct forelimb positioning. This is supported by the pharmacological perturbations of the levels of RA signaling, in which RA treatment shifts the posterior boundary of Hoxb4 to more posterior, while RA inhibitor treatment advances the entire limb position to more anterior (Moreau et al., 2019a).

Hindlimb positions also vary between avian species. 5’ Hox genes, including Hox9-13, are activated by a secreted factor Gdf11. The inhibition of Gdf11 activity using a pharmacological drug perturbs posterior Hox gene expression and results in a posterior shift of the hindlimb position in the chicken embryo. A comparative analysis revealed that the timing of Gdf11 expression varies among the bird embryos, such as Chick, Quail and Emu. Moreover, time of Gdf11 expression correlates very nicely with the hindlimb positions in these birds, suggesting that the shifts in Gdf11 expression may play a role in the variation seen in hindlimb positioning. Noticably, this correlation is not restricted to avian species, but is also seen in other tetrapods including African Clowed Frog, Chinese soft-shelled turtle, Ocelot gecko and Japanese striped snake (Matsubara, et al., 2017), suggesting that the timing of Gdf11 expression may play a universal role in regulating tetrapod hindlimb positioning.

## Timing the specification of the proximal to distal axis

**FIGURE 2:**
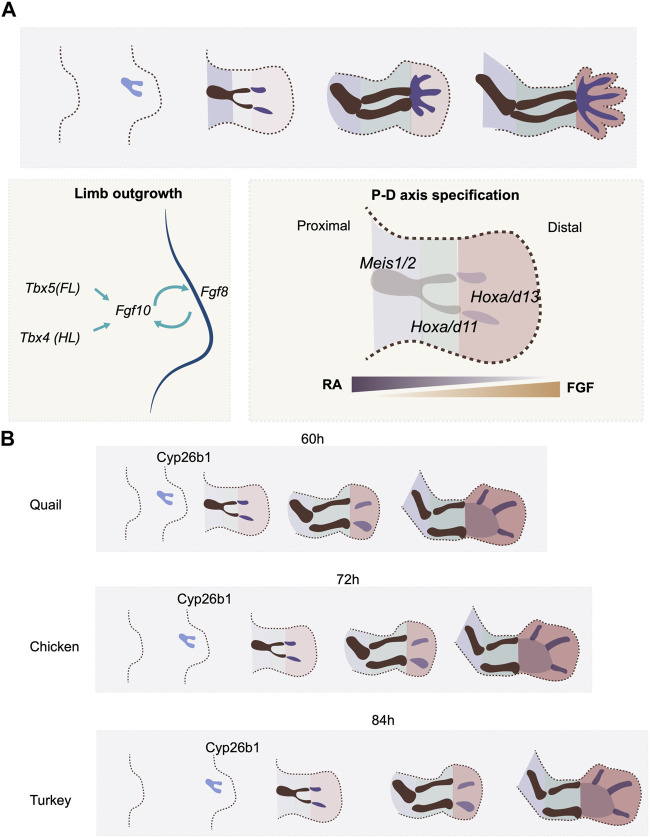
P-D axis specification and temporal alterations. **(A)** P-D axis is specified by reciprocal gradients between RA and FGF signaling. Three segments are specified along the P-D axis, marked by the expression of transcription factors, Meis, Hox9 and Hox11. **(B)** the timing of expression of a RA degradation enzyme. Cyp26b1, regulates the timing of P-D axis specification rate in quail, chicken and turkey embryos.

The proximodistal axis of the limb is organized through the activity of two signaling centers, the AER producing Fgf10 distally and the flank producing RA proximally. The Fgf10-Fgf8 positive feedback loop is the most active in the distal end of the limb bud underneath the AER, from which it decays progressively towards the proximal end (Mariani et al., 2008). By contrast, RA is synthesized and emanates from the trunk, from which it diffuses distally. The distal region also expresses the RA degrading enzyme, Cyp26b1, which helps shapes the RA gradient from proximal to distal (MacLean et al., 2001). The anti-parallel gradients between FGF and RA create varying positional inputs to cells along the PD axis, ultimately establishing three distinct segments marked (from proximal to distal) by transcription factors Meis1/2, Hoxa11, and Hoxa13, respectively (Boulet and Capecchi, 2004) (Cunningham et al., 2013) (Fromental-Ramain et al., 1996) (Mariani et al., 2008) (Mercader et al., 2000; Tabin and Wolpert, 2007) (Figure 2A). The three regions later become the stylopod, (the proximal limb segment, extending from the shoulder/hip to the elbow/knee), zeugopod (the middle limb segment, extending from the elbow/knee to the wrist/ankle), and autopod (the distal limb segment).

### Kinetics to establish the RA gradient regulates the timing of proximodistal axis specification

Natural variation in the timing of proximodistal axis specification in the limb bud is seen between quail and chick embryos. The embryonic development of the two avian species is highly similar at early stages. Indeed, because of this, early interspecies grafts can be successfully used in lineage analyses (Teillet et al., 1999). However, the gestation time of the quail is shorter than that of the chick (16 days vs. 21 days). This indicates that heterochronic changes must be present between the two species in certain, if not all, developmental processes. A recent study shows that there are, indeed, heterochronic changes in limb bud development between these two species. Both species initiate forelimb development 3 days after egg incubation with a thickening of the limb field. However, the maturation of skeletal patterning occurs 12 h faster in the quail embryo (60 h) than in the chicken (72 h) (Stainton and Towers, 2022) (Figure 2B). This faster limb developmental rate in the quail embryo involves a higher rate of cell proliferation, the earlier expression timing of proximodistal axis patterning genes such as Hoxa11 and Hoxa13, and finally, the early expression of the chondrogenesis factor Sox9 (Stainton and Towers, 2022). Although the expression of Fgf8 can be detected in the AER before the onset of Hoxa11 and Hoxa13 expression, the RA degradation enzyme Cyp26b1 appears earlier in quail than in chick (Stainton and Towers, 2022) (Figure 2B). As RA is already produced in the flank prior to the time of limb initiation, the earlier expression of Cyp26b1 would shape the RA gradient in the limb bud precociously in the quail as compared to the chick, which would hence be expected to initiate proximodistal patterning at an earlier stage. To test this hypothesis, the quail wing bud was treated with continuous RA at the onset of development. The treatment effectively delayed the onset of Hoxa11 and Hoxa13 expression. Moreover, the growth phase of the treated wing bud was extended compared to the regular quail limb bud, making it resemble the timing of the chicken wing (Stainton and Towers, 2022).Therefore, the prolonged, higher level of RA signaling allowed the developmental timing in the quail wing to recapitulate that of the chicken (Stainton and Towers, 2022).

This RA-mediated mechanism for controlling the time scale of proximodistal axis specification appears to be used in a variety of avian species. The turkey embryo develops for a longer period (28 days) than the chicken. Accordingly, the turkey wing bud develops more slowly than in the chicken, including slower cell proliferation and delayed onset of Hoxa11 and Hoxa13 expression (Figure 2B). Consistent with the result in the quail limb bud, treating chicken wing buds with RA results in the slowdown of limb bud development, including the rate and duration of tissue growth, and the timing of patterning gene expression. The resulting overall proximodistal axis developmental rate becomes more similar to that of the turkey (Stainton and Towers, 2022). These results thus support a model where the timescale of proximodistal patterning of the limb bud is controlled, at least in part, by the rate at which the RA gradient is established, via modulation of the timing of expression of the RA inhibitor Cyp26a1. What regulates the expression timing of Cyp26a1 is still to be determined. As the rate of development of the limb bud aligns with the overall gestation time across the three species, one cannot rule out the possibility that a general scaling mechanism is involved in the regulation of the timing of Cyp26a1 expression. This study also provides an elegant example of how the trade-off between tissue size and differentiation rate can be achieved, in this case, by a patterning signal. Such growth-differentiation trade-offs are fundamental at the evolutionary scale, as larger animals tend to have slower developmental rates (Blueweiss et al., 1978).

## Heterochrony and digit number

The evolutionary loss of digits has occurred repeatedly in tetrapod lineages. In a classic study, Alberch and Gale (1985) (Alberch and Gale, 1985) put this evolutionary pattern into a developmental context. First, they established the order in which digits form during ontogeny in both frogs and salamanders, and found that the sequence of digit formation was different in these two clades. They then categorized the evolutionary order in which digits were lost in each group. They found an inverse relationship where, in both frogs and salamanders, the first digit to be lost evolutionarily, was the last digit to form developmentally (in the pentadactyl species). Moreover, they showed that experimental reduction of the limb bud size, using mitotic inhibitors, yielded the same pattern of digit loss as seen in the variation between natural population (first digit lost corresponding to the last one to form developmentally) (Alberch et al., 1985). This was an important study for establishing the concept of “developmental constraint” (there being a constraint on the order in which reduced digits could be selected). Mechanistically, in the absence of mitotic inhibitors, the authors suggested two ways in which smaller limb fields could have occurred, through “global developmental truncation” (corresponding to progenesis) or through “a slowdown in the rate of proliferation” (corresponding to neoteny), both modes of heterochrony. But how this is actually achieved at a developmental level had to wait until the genetic mechanisms of digit specification were at least partially worked out.

### Digit specification

Pioneering work by Saunders et al. revealed the crucial role of a specialized region located at the posterior of the limb bud in determining the anteroposterior axis. Transplantation of this region to the anterior margin of another early limb bud produced a mirror-image duplication of the digit arrangement (Saunders, 1968). This posterior region was subsequently named “zone of polarizing activity” (ZPA). A model was formulated whereby the ZPA secretes a morphogen signal which forms a gradient from the posterior to anterior region, conferring digit identity as a function of distance from the ZPA (Wolpert, 1969). The ZPA-derived patterning molecule was later revealed to be Sonic Hedgehog (Shh). The Shh expressing region of the limb bud is congruent with that of the ZPA, and the transplantation of Shh-expressing cells to the anterior margin faithfully recapitulates the mirror-image digit duplication phenotype produced in the ZPA transplantation experiments (Riddle et al., 1993). Conversely, loss of Shh function in the limb bud results in a loss of digits with the exception of a single dysmorphic anterior digit, in both mice and chicks (Chiang et al., 2001; Ros et al., 2003).

The mechanism by which Shh patterns the digits has been a subject of intense study for more than 25 years. Yet the issue remains unresolved, with somewhat contradicting evidence from experiments in the chick and mouse, and it is currently controversial whether Shh acts directly on digit primordia, indirectly through a secondary signal, or a combination of both; as well as whether it is the levels of Shh activity, the duration of exposure, or both that is important (see review by McQueen and Towers, 2020, and the recent publication by Zhu et al., 2022) (McQueen and Towers, 2020; Zhu et al., 2022). Weighing the various lines of evidence for these important issues is beyond the scope of this review on heterochrony. Rather, in the current context, it is important to bear in mind a few, well accepted, general aspects of the role of Shh in digit specification. First, whether acting directly or indirectly, Shh is the key upstream signal responsible for patterning the digits, i.e., giving their primordia distinct identities such that they form digits with the proper anatomical structures. Second, specification of digit identity is independent of the determination of the number of digits that will form. For example, in the absence of digit type specification, if anterior limb bud cells are disaggregated, mixed, then pelleted and grafted into a host embryo in an ectodermal jacket (a so-called “recombinant limb”), an un-patterned array of digits are formed (Zwilling, 1964; Pautou and Kieny, 1973). The repeated pattern of digit—non-digit appears to be established through a Turing-like self-organizing system (Wilby and Ede, 1975; Newman and Frisch, 1979; Raspopovic et al., 2014), with the width of the limb bud providing a boundary condition in the Turing pattern, determining the number of digits. Third, Shh appears to play a critical role in this as well, stimulating the anterior-posterior expansion of the limb bud both through the regulation of pro-proliferative Fgf factors in the AER (Laufer et al., 1994; Niswander et al., 1994) and through regulation of the GI-to-S cell cycle transition in the mesenchyme (Towers et al., 2008). Finally, the two roles of Shh (triggering the cascade of events leading to digit identity and in promoting expansion of the digit-forming region), appear to be distinct and separable activities (Zhu et al., 2022). There is some genetic evidence that the patterning phase of Shh activity could be extremely short, in the order of a few hours (Zhu et al., 2022), although other studies suggest that it could be somewhat more prolonged (Towers et al., 2011). In either case, it is clear that the proliferative effect of Shh, and its role in providing sufficient substrate to form the full set of digits, extends temporally well beyond the role of Shh in digit specification (Zhu et al., 2022).

### Shh and digit reduction in skinks

Like frogs and salamanders, discussed above, some groups of lizards have undergone evolutionary reduction in the number of their digits. For example, the Australian Squamata Genus Hemiergis has distinct populations (falling into three species) with 5, 4, 3, or 2 toes. Shapiro (2002) examined the ontogeny of the limbs in each of these groups. As in the frogs and salamanders analyzed by Albech and Gale, the digits of the five-toed species are initiated in a defined order (Alberch and Gale, 1985). Moreover, the order in which the digits are lost evolutionarily in related groups is indeed in the reverse order of their induction during limb development. Importantly, in species with reduced number of digits, the digits that do form have just as many phalanges as seen in the corresponding digits of the 5-toed form. Strikingly, however, all the digits in Hemiergis begin their morphogenesis in a very quick sequence. Thus, the first digit to be initiated is still in the process of forming additional phalanges when the last digit begins its morphogenesis. As such, one cannot generate a reduced number of full-sized toes simply by truncating developmental processes prematurely. For example, to generate a 4-toed form, in principle, digit formation could be stopped just before the last-forming toe was initiated. However, that would result in the toes that did form being incomplete, with fewer phalanges than in the ancestor. In other words, at an anatomical level, there is not a simple heterochronic mechanism underlying the evolutionary sequence.

This opinion changes, however, when viewed at a genetic level. In a subsequent study (Shapiro et al., 2003), it was found that the duration of Shh expression is shortened in Hemiergis species with reduced numbers of digits, correlating with decreased cell proliferation. In light of our knowledge of Shh function, discussed above, the pattern of digit loss becomes understandable. Shh activity in the limb bud specifies five distinct digit identities in all populations. Subsequent Shh exposure of various duration determines how much the limb bud expands and hence how many digits can form. However, as all the digits are fully specified, those that do form have a complete structure.

Indeed, it was on the basis of this analysis that Shapiro et al. were the first to postulate an early role for Shh in specifying limb identity, and a later one in maintaining cell proliferation and survival (Shapiro et al., 2003), a model subsequently confirmed genetically in mice (Zhu et al., 2008). Thus, at a genetic level, the evolutionary loss of digits in hemiergis is heterochronic, being driven by a change in the length of time Shh is expressed, and in particular by differences in the time at which its expression ceases. This, in turn, begs the question of what controls the termination of Shh activity in the limb bud. Unpublished analysis of the known cis-regulatory sequence that controls Shh limbs expression (the ZRS - discussed more fully below) failed to identify any differences between different Hemiergis populations (Shapiro, personal communication), shifting the focus to extrinsic factors potentially modulating the duration of Shh expression.

### Termination of Shh expression

**FIGURE 3:**
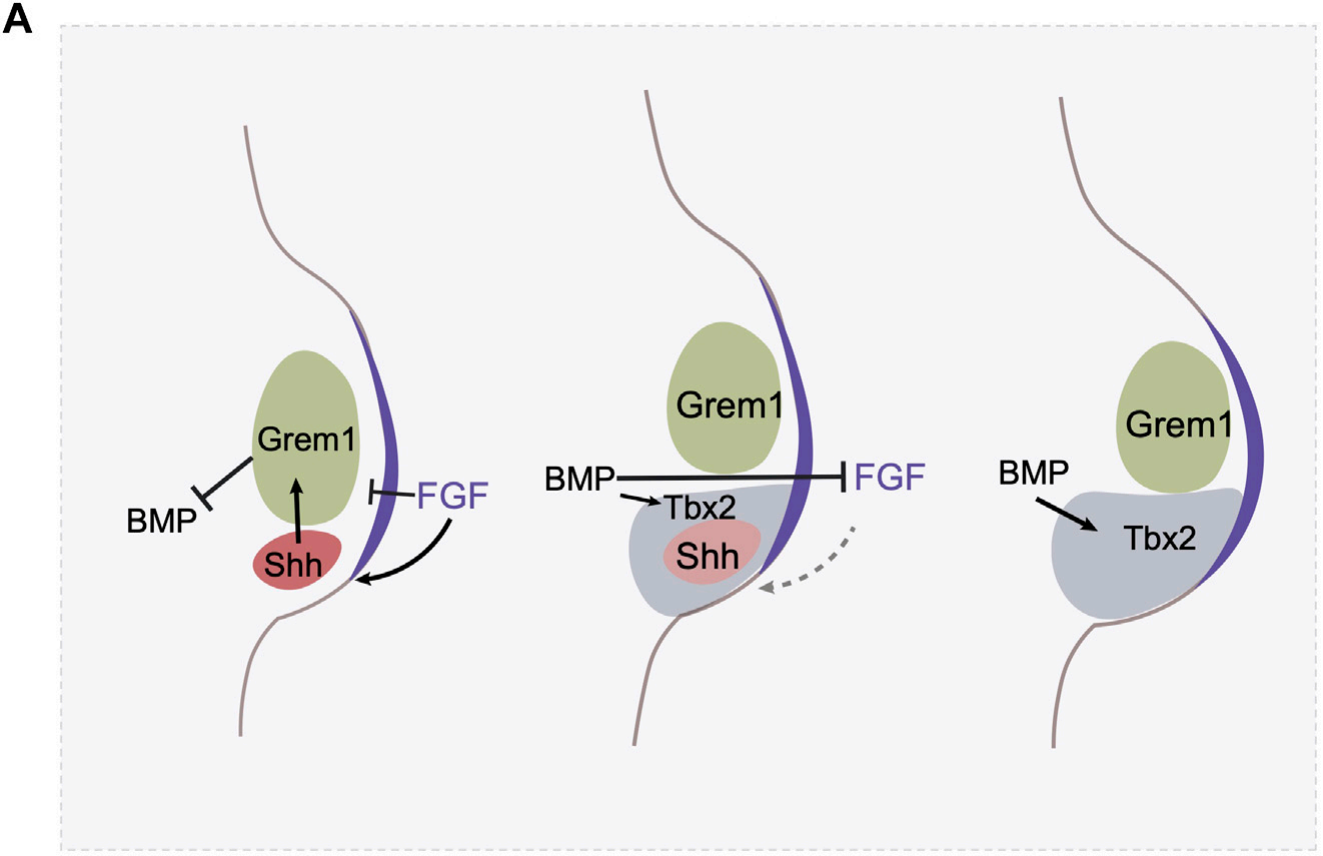
The termination of Shh expression involves multiple mechanisms. **(A)** The AER derived FGF signaling maintains Shh expression, and BMP signaling antagonizes FGF signaling in the AER. Shh activates Gremlin in the mesenchyme, which prevent BMP’s antagonistic effect on AER. In the meantime, FGF at AER prevents Gremlin expression. In the initial stage, the FGF-Shh-Gremlin loop is strong enough to maintain the FGF-Shh loop. Shh expression triggers cell proliferation and the digit identity specification. However, as the limb bud expands, BMP activates the expression of a transcription factor Tbx2 in the posterior region which antagonizes Gremlin’s expression. Gremlin at this region is further hampered by the high level of FGF activity. As a result, these negative feedback loops allow the creation of a spatial gap between ZPA and Gremlin expressing domain and represses the FGF-Shh loop and triggers the termination of Shh expression.

Shh expression in the posterior limb bud requires continued exposure to Fgf activity emanating from the AER (Laufer et al., 1994; Niswander et al., 1994). This relationship is reciprocal, Shh and AER-derived FGF signaling forms a positive feedback loop to sustain proximodistal and anteroposterior outgrowth. This positive feedback loop is interfered with by the ventralizing signal Bmp, which downregulates expression of Fgf family members in the AER (Zuniga et al., 1999; Bastida et al., 2009). Shh, however, induces the expression of a Bmp antagonist, Gremlin in the adjacent mesenchyme, preventing Bmp activity from antagonizing Fgf production. The Shh-Gremlin-Bmp negative feedback loop is initially strong enough that it ensures continuous outgrowth of the limb bud and maintains Shh expression (Khokha et al., 2003). As the ZPA region expands, the descendants of the Shh-expressing cells are pushed by mass action to the anterior, outside of the ZPA, and gradually lose Shh expression. Shh-expressing cells and their descendants are refractory to expressing Gremlin (Scherz et al., 2004; Farin et al., 2013). This creates a spatial barrier: the source of Shh is progressively separated from cells that can respond by activating Gremlin expression, until the responsive domain is too far away, Gremlin is not induced, and hence Bmp activity is no longer inhibited. As a consequence, Fgf expression is repressed by Bmp signaling, thereby terminating Fgf—dependent Shh expression. The reason Shh-expressing cells, and their descendants, are refractory to Gremlin induction has also been elucidated. Bmp acts, in the posterior limb bud, to induce the expression of the transcription factor Tbx2, which represses Gremlin expression in the ZPA and in the expanding population of cells derived from the ZPA (Farin et al., 2013) (Figure 3A).

The Shh-Fgf feedback loop is attenuated through a second mechanism as well. Fgf signaling itself, high levels, represses Grem1 expression (Verheyden and Sun, 2008). Thus, the Shh/Fgf positive feedback loop drives outgrowth but concomitantly results in increasing levels of Fgf signaling, until it reaches a threshold where it triggers the Fgf/Grem1 inhibitory loop (Figure 3A). Both of these feedback mechanisms create self-terminating circuits, the integration of which regulates the duration of Shh activity in the limbs. In addition, heterochronic graft experiments of the ZPA region between young and old limb buds where Shh is expressed at high and low levels respectively, indicate that Shh expression is also regulated by a cell-intrinsic timer, the nature of which remains unknown (Chinnaiya et al., 2014). How these regulatory feedback loops might differ between different species of Hemiergis in modulating the duration of Shh expression remains to be explored.

## Limb loss in snakes

While cis-regulatory sequences upstream of Shh activity do not seem to be involved in the evolution of digit loss in skinks, they do appear to have played a central role in the evolutionary loss of the entire limb in snakes. Snake-like body plans evolved multiple times in the squamate lineage. In some snake-like lizards the forelimbs are lost, in others the hindlimbs are lost, whereas some lineages such as the glass lizard are completely limbless, like snakes themselves (Brandley et al., 2008).

The evolutionary loss of a structure, such as the limb, in and of itself, would not necessarily be viewed as a heterochronic change. This applies to the loss of the forelimb in snakes, which is believed (based on the fossil record) to have preceded the loss of hindlimbs (Apesteguia and Zaher, 2006). Neither fossil extinct snakes nor extant forms display any remnant of pectoral appendages, nor are there any known cases where a modern snake embryo initiates a forelimb bud (Raynaud, 1985). The forelimb has simply been lost, and how this may have occurred is still poorly understood.

In contrast, there are vestiges of the hindlimb in both fossil snakes and occasionally in modern basal snakes (boas and pythons), although the hindlimb is completely missing in more advanced snakes. Adult pythons and boas retain a pelvic girdle as well as a variable remnant of a femur. However, embryonically all individuals of these clades form transient limb buds that form chondrogenic condensations corresponding to the tibia, fibula, and footplate in addition to the femur, prior to degenerating (Leal and Cohn, 2016). Thus, hindlimb loss in basal snakes corresponds to a truncation of a developmental process, or progenesis, a form of heterochrony. Advanced snakes appear to have taken this one step further, truncating the limb development program at its onset. At a molecular level these evolutionary changes in hind limb formation appear to have been achieved through the sequential degradation of the cis-regulatory sequences controlling Shh expression in the limb bud.

Specific expression of Shh in the posterior limb bud is regulated by the ZRS (ZPA Regulatory Sequence) enhancer, located within an intron of another gene, Lmbr1, more than 1 Mb away (Lettice et al., 2002). Deletion of this sequence results in a complete absence of Shh expression in the limb (Sagai et al., 2005), while point mutations within it can result in anterior ectopic Shh expression and polydactyly; or reduction in, or even ablation of, Shh expression (Lettice et al., 2008). These results are explained by the fact that the ZRS has a dual function, and corresponding bipartite organization, where the 5’ domain of the ZRS is responsible for the proper spatial and temporal activation of Shh in the posterior limb bud, while the 3’ domain is important for the looping of chromatin to the Shh promoter and is necessary to prevent inappropriate activation of Shh in the anterior (Anderson et al., 2014).

Genomic comparison indicates that there have been multiple mutations within the ZRS in the python lineage (Kvon et al., 2016; Leal and Cohn, 2016). Tests of python, and limbed lizard ZRS activity were carried out with reporter constructs in transgenic mice and show that while the lizard ZRS drives robust activity in the mouse ZPA, the python ZRS is much weaker, and drives expression in only a small subdomain of the ZPA (Leal and Cohn, 2016). Moreover, when the mouse ZRS is replaced by the python ZRS, using CRISPR-Cas9 technology, the resultant mice exhibited limb truncation highly reminiscent of the structure of the python hindlimb (Kvon et al., 2016). The mechanistic basis by which the python mutations alter Shh expression have not been formally tested, but the ZRS changes in python include mutations in putative binding sites for key Shh-regulating transcription factors, including HoxD, ETS, and Hand1 binding sites, potentially explaining their altered expression profile (Kvon et al., 2016; Leal and Cohn, 2016).

In advanced snakes, the heterochronic truncation of the limb development program is pushed even further back to the initiation of the limb bud. In these animals the ZRS is barely identifiable, as the enhancer is much more degenerated than in pythons and boas (Kvon et al., 2016; Leal and Cohn, 2016). The alterations of the ZRS may have been critical in the evolution of total limb loss in advanced snakes, as it seems to have been in the limb reduction in pythons and boas. Alternatively, the complete loss of limbs could have been achieved by a different mechanism, and the subsequent degeneration of the ZRS simply reflecting neutral mutation in the context of a lack of functional selection to maintain the enhancer once limb buds were lost.

## Hyperphalangy

The evolution of digit reduction in Hemiergis and hind limb loss in basal snakes can both be viewed as having arisen through paedomorphosis, the truncation of developmental trajectories. Limbs have also evolved through the opposite mode of heterochrony, peramorphosis, or the extension of developmental trajectories. An example of this is the evolution of hyperphalangy, or an increased number of phalanges, in the digits. Although relatively small increases in the number of phalanges are occasionally seen in terrestrial tetrapods, dramatic hyperphalangy has repeatedly evolved in the flippers of secondary aquatic taxa (those descended from land-dwelling species) such as extinct ichthyosaurs, mosasaurus, and modern cetaceans (whales and their relatives) [reviewed in (Fedak and Hall, 2004)].

Key to understanding hyperphalangy is the recognition that the individual autopod phalanges do not have specific developmental identities. More proximal skeletal elements such as the humerus, and radius and ulna, can be viewed as having distinct identities, being defined by the expression of selector genes, such as Meis1/2 in the stylopod, and Hoxa11 in the zeugopod. However, the autopod elements are not specified individually, but rather are generated through a cyclic program of chondrogenesis and segmentation (Richardson et al., 2004), with every species having defined developmental parameters specifying the ratio of decreased size from one phalanx to the next within a digit (Kavanagh et al., 2013). Hyperphalangy, thus, results from the prolonging of the reiterative program of phalanx generation (peramorphosis).

Prolonging the process of phalanx generation in a digit, with a consequent generation of an extra segment, can be achieved experimentally through manipulations that maintain expression of Fgf8 in the AER overlying a forming digit (Sanz-Ezquerro and Tickle, 2003). Based on this, it was proposed that duration of Fgf activity emanating from the AER, coupled with a characteristic periodicity of segmentation, is responsible for establishing the appropriate number of phalanges in each digit. Consistent with this model, hyperphalangy in cetaceans is accompanied by specific maintenance of the AER (Richardson and Oelschlager, 2002), and Fgf8 expression (Cooper et al., 2007), above the digits forming additional phalanges (and not above adjacent digits that do not exhibit hyperphalangy).

## Heterochrony in the timing of forelimb and hindlimb development

The time of limb bud development is not always congruent in forelimb and hindlimb. In fact, one might expect that the hindlimb should develop somewhat later than the forelimb, as the timing of posterior Hox gene expression is later than that of the anterior Hox genes (Deschamps and Duboule, 2017; Moreau et al., 2019b). However, examination of embryogenesis in different phylogenies shows that this is not always the case. A relative delay in formation of the posterior appendage relative to the anterior appendage is indeed observed between the rostral and caudal fins in zebrafish, and the fore- and hind limbs of certain types of lizards and mammals. However, in most bird embryos, the formation of forelimb and hindlimb buds appears almost simultaneously; and in amphibians, the hindlimb structures, which develop before metamorphosis, are more advanced than the forelimb, the outgrowth of which only becomes apparent after metamorphosis (Richardson et al., 2009; Royle et al., 2021). This indicates multiple aspects of heterochronic regulation, exceeding the scale that can be accounted for by variation in timing of Hox expression. When referring to the timing of limb development, several aspects must be considered, including the time of initial formation of the limb buds, the time to establish a fully matured skeletal pattern, and the growth rate in the post-patterning process.

**FIGURE 4:**
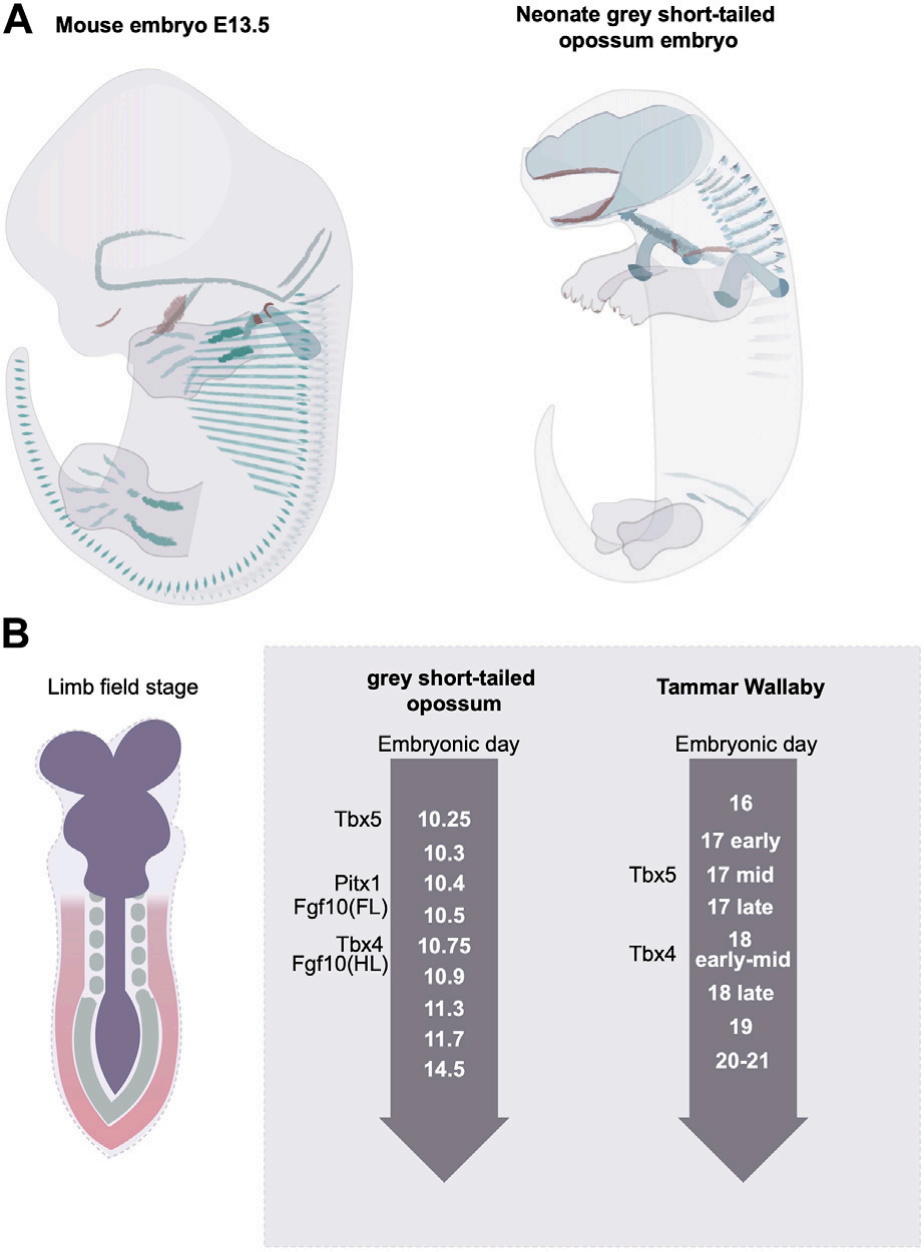
Rostral to caudal developmental heterochrony in mammalian embryos. **(A)** the grey short-tailed opossums display a severe rostral-to-caudal heterochrony marked by the reduced size in hindlimb and the lack of matured cartilage structure at the rostral end. Green indicates the differentiated cartilage and red labels the ossified bone structures. **(B)** The marsupial embryos display limb developmental heterochrony at the limb initiation stage.

At the initial phase of limb bud development, nearly all mammalian clades display a delay in the hindlimb outgrowth compared with the forelimb (Richardson et al., 2009; Werneburg and Sánchez-Villagra, 2011). In the non-placental mammals, such as marsupials, where birth is altricial, the delayed hindlimb growth is the most severe (Richardson et al., 2009). The level of heterochrony is species-specific. In the grey short-tailed opossum embryo, for example, at birth, the hindlimb has an established pattern of digits, but the overall size is reduced, and the cartilage structure is poorly differentiated (Keyte and Smith, 2010; Smith and Keyte, 2020) (Figure 4A). The fat-tailed dunnart, on the other hand, shows a more extreme hindlimb retardation where at a time of birth the hindlimb remains in an undifferentiated limb bud state. In the case of the grey short-tailed opossum, the hind limb expression of Tbx4 appears more than half a day later than Tbx5 in the forelimb indicating that the heterochronic delay is already present at the limb initiation stage (Keyte and Smith, 2010). A similar scale of delay can be found in another marsupial species, the Tammar Wallaby (Chew et al., 2014) (Figure 4). Interestingly, in grey short-tailed opossum embryos, the anterior-posterior heterochrony not only exists in limb development but also in somitogenesis; a 4-fold time difference has been identified for anterior vs. posterior somite generation rate, and the newly born neonates lack differentiated cartilage in the posterior somites (Keyte and Smith, 2012). It is worth noting that the length of the tail of the grey short-tailed opossums appears similar to that of the mouse at the time of birth, therefore it is likely that the heterochrony of somitogenesis not only comes from the delay of posterior somite generation but also from an acceleration in anterior somite development.

Another interesting aspect of the heterochrony in marsupial embryos is the compensatory growth of hindlimb after the onset of lactation. After the embryos are born and can access the milk in the pouch, the hindlimb grows and differentiates quickly and it soon catches up to the stage of forelimb (Cook et al., 2021). It is possible that this acceleration of hindlimb growth is regulated by a nutrient sensing mechanism. Indeed, an “energy trade-off” hypothesis has been postulated to explain the anterior-posterior heterochrony of marsupial embryos, as the level of growth heterochrony correlates inversely with the length of the gestation time (Smith and Keyte, 2020). The post-natal growth rate seems to be regulated by maternal milk supply as the cross-fostering between young and old age neonates allows the growth rate to adjust to the new maternal lactation age (Trott et al., 2003; Kwek et al., 2009). It is unknown whether it is the presence of the nutrient supply that stimulates the hindlimb-specific increase in growth or whether other hormone-related factors come into play. It is also worth noting that different aspects of heterochrony may be regulated by distinct mechanisms, such that the heterochrony in limb initiation may not be regulated by the same mechanisms as those for post-patterning. Overall, the heterochrony in anterior and posterior development, partially manifested by the timing differences needed to develop forelimb and hindlimb appendages, is a fascinating question and yet the molecular and cellular basis has been poorly explored. The understanding of mechanisms accounting for this striking heterochronic phenotype will be instrumental to understanding the regulation of the rate of growth and differentiation.

As noted above, limb bud formation begins with localized EMT in the presumptive limb fields, triggered by expression of Tbx5/4 (Gros and Tabin, 2014). It therefore makes sense that the heterochronic delay in hindlimb development seen in marsupials would be controlled at the very first step of Tbx4 expression. However, this does not turn out to be the case in all examples of developmental delay in limb initiation. The wing of the flightless emu is greatly reduced in size, in part due to a delay in the outgrowth of the wing buds. Surprisingly, however, a recent study showed that the initial steps of forelimb development, including Tbx5 activity and consequent EMT, occur at equivalent stages in the emu and chick (Young et al., 2009). Rather, it is a subsequent step in the process, the induction of the downstream gene FGF10, that is modulated, resulting in a failure to activate target genes necessary for proliferation of the limb mesenchyme.

## Conclusion and perspectives

The changes in time, tempo, or duration of developmental processes are a prevailing phenomenon during evolution. There is a long history of characterizing developmental heterochrony, yet mechanistic insight is only beginning to emerge. Understanding heterochrony requires an understanding of the genetic and cellular transformations that shape tissues. As a comprehensive developmental system where, genetic drivers are largely revealed and cellular events are characterized, limb development is one of the best-suited systems to study how timing variations cause morphological adaptations and to yield principles of how timing mechanisms can drive changes in tissue formation. As supported by the many examples discussed here, timing mechanisms have been extensively used in establishing the correct pattern of the limb. As a result, evolutionary cases can be found in which alterations in timing cause different anatomical changes in the limb. Many of the case studies we discuss here essentially reveal the molecular or cellular basis of the heterochronic events. However, a systematic solution is still needed to fully comprehend how heterochrony causes the observed phenotypes.

Understanding the precise modulation of the timing of gene expression is still a daunting task, and currently the phenotypes that result from temporal perturbations of non-linear interactions are difficult to interpret. The development of new quantitative genetic approaches, advances in imaging, and modelling hold the potential to better solve the problems. In addition, although many limb heterochrony issues have been addressed, there are still many that await investigation. Examples include the various aspects of anterior-posterior heterochrony in mammals and the heterochrony in chondrogenesis in different segments of the limb, which have been previously reviewed (Richardson et al., 2009). Moreover, in nearly all the cases we discuss here, heterochrony results from alterations in gene expression. The extent to which environmental adaptation or physiological changes also contribute to the timing of tissue development will be an interesting topic to explore.
